# Cyberspace as a new arena for terroristic propaganda: an updated examination

**DOI:** 10.1007/s10202-012-0108-3

**Published:** 2012-08-09

**Authors:** Elizabeth Minei, Jonathan Matusitz

**Affiliations:** 1 University of Oklahoma, Norman, OK USA; 2 Seminole State College, University of Central Florida, Partnership Center (#UP 3009), 100 Weldon Blvd., Sanford, FL 32773 USA

## Abstract

This paper analyzes the role of propaganda use in cyberterrorism. The main premise is that cyberterrorists display various semiotic gestures (e.g., the use of images and Internet videos) to communicate their intents to the public at large. In doing so, they communicate themes—these themes range from hate to anger. Cyberterrorism, then, is a form of theater or spectacle in which terrorists exploit cyberspace to trigger feelings of panic and overreaction in the target population. In many cases, this form of propaganda is the primary means of communication for various cyberterrorist groups to convey their intents. Internet sites also produce numerous opportunities for in-group communication and publicity.

## Introduction

In this paper, the role of propaganda use in cyberterrorism is being analyzed. The main premise is that cyberterrorists display various propagandist gestures (e.g., through the use of images and Internet videos) to communicate their intents to the public at large. In doing so, they communicate themes—these themes range from hate to anger. Cyberterrorism, then, is a form of theater or spectacle in which terrorists exploit cyberspace to trigger feelings of panic and overreaction in the target population. In many cases, this form of propaganda is the primary means of communication for various cyberterrorist groups to convey their intents. Internet sites also produce numerous opportunities for in-group communication and publicity. This analysis fills a gap in research on both propaganda and cyberterrorism in that the vast majority of studies on terrorism propaganda, so far, have focused on traditional terrorism, but very little on cyberterrorism (Steuter and Wills 2009).

In this analysis, the first section offers general perspectives on cyberterrorism. As such, the authors describe cyberterrorism, its origins, and the various forms and techniques used by cyber attackers. Also provided in this section is a short explanation of the function of semiotics in cyberterrorism. What comes subsequently is the heart of the present analysis: an examination of propaganda use in cyberterrorism. It begins with historical perspectives of propaganda use; then, it delves into specific cases of propagandist gestures with respect to cyberterrorist acts. For instance, following the London bombings in 2005, “The Martyrdom Will of Mohammad Sidique Khan” became a viral video launched by a Muslim cyberterrorist group. Another example is that of Irhabi007, an attacker playing cat-and-mouse games with authorities through his websites. The next-to-last section of this analysis examines a case study of cyberterrorist propaganda—specifically, a propagandist act of the World Fantabulous Defacers (WFD)—by using semiotics and explains how the cyberterrorist act works both as a symbol and as terrorism. This analysis ends with a discussion section that also offers suggestions for future research.

### General perspectives on cyberterrorism

This section describes cyberterrorism, the origin of the word and the various forms and techniques used by cyber attackers. It also provides a short explanation of the function of semiotics in cyberterrorism.

#### Cyberterrorism: definition

In order to understand the full scope of how destructive and powerful cyberterrorism can be, it is important to gain a basic understanding of the actual word. The word “cyberterrorism” comes from the portmanteau of “cyberspace” (i.e., the makeup of data, algorithms, and computer networks) and “terrorism” (i.e., premeditated, politically motivated violence committed against innocent persons or noncombatants) (Conway 2002; Deutsch 1997). Cyberterrorism, in and of itself, is a method of attack designed to damage, tamper with, or destroy critical points of national infrastructure by controlling and manipulating computer networks (Denning 1999, 2000; Libicki 2009; Sloan 2006). The prefix “cyber” suggests that this type of terrorism occurs throughout cyberspace and is, in turn, accessible through computers (Conway 2002). The basic premise of traditional terrorism is the threat, or the actual use of violence against people or property, with the intention of inflicting enough harm to garner attention, create fear, and influence decision-making (Sloan 1981). A different concept than conventional crime, terrorism has roots in strong ideological motives, often with a goal of imposing principles and beliefs by illegal and violent means (Axelrod and Nicoletti 2009).

Though most instances of cyberterrorism occur through Internet use, it is important to recognize that the lesser utilized mechanisms of the telephone also play a role in conducting denial-of-service attacks (i.e., D.O.S. attacks), which render computer networks inaccessible, inoperable, or ineffectual, thus easing the transmission and distribution of propaganda by the attacker (Howard 2009). One such example of a D.O.S. attack would be a victim who is injured attempting to get help by dialing 911, only to be met with continuous dropped phone calls or just a dead line (Brown 2006). In causing attacks, a cyberterrorist has access to any given nation vulnerable to attacks of a grand scale. What this means is that irreparable damage can be caused due to a nation’s heavy reliance on critical infrastructure that is rooted in computer networks (Lewis 2002). Using a universal weapon as seemingly harmless as the computer, cyberterrorists have at their fingertips a medium that allows them to cause great damage with minor consequence (Gorge 2007). Files can be stolen and corrupted, computer viruses can be spread, and these are all due to the easy access provided by the Internet. In some cases, a multiplied threat exists when the attacker is a former employee, familiar with the computer network, and wishing to cause harm (Misra 2003). The destruction of websites, knowingly crashing selected networks, causing denial of service in crisis situations, spreading malicious computer viruses, causing physical destruction and tampering with financial interactions, all while inducing panic and causing psychological harm to targets, are all utilized methods commonly known as information warfare (Paul 2008).

This form of attack holds greater appeal than that of the conventional methods used in the past for many reasons. For example, the costs of such an attack greatly diminish when, all things considered, the equipment needed for such an attack does not go beyond that of a computer and an online connection rather than the traditional weapons of guns or bombs used in terror situations of the past (Weimann 2005). Previous examples of traditional terrorist attacks (carried out in real time) required massive amounts of organized locations in which attackers utilized software such as robotic networks that globally hijack any number of targets and render them helpless (Aaviksoo 2008). It is precisely this lack of physical presence in regard to a target that provides a foundation for the rationale behind why cyberterrorism is a preferred method.

A high level of anonymity comes with a lack of borders, barriers, and authority that leaves an attacker virtually without consequence to target anyone or anything across the globe (Weimann 2005). This notion reflects the idea that crimes committed via computers are of a global nature in which unleashing worms and viruses that steal information are not limited on a small scale, but can occur between entire countries and nations when attackers are given free rein to commit crimes internationally, against individuals, corporations, and governments (Cassell 2006). Western infrastructures have been a primary target; so have highly populated areas, which will remain primary venues that become susceptible to attacks (Gunaratna 2005). Combined with the notion that cyberterrorism is both inexpensive and anonymous, as well as remote, an attacker is not forced into physically demanding high-risk situations; nor do they have to be as crafty to outwit security systems (Weimann 2005).

The rationale for the occurrence of cyberterrorism has included that of political motivation (Baudrillard 2002). When emblematic western infrastructures such as banks, hotels, and utilities are considered, the sheer volume of targets becomes endless, causing the focus for an attacker to switch to a strategic nature, where the motivation for an attack is fueled by the amount of damage that can be done (Gunaratna 2005). An appealing factor in the equation of cyberterrorism is that the attacks are conducted from a location removed from the target (Weimann 2005). An attacker can handpick a target based on vulnerability in various areas of government, health, commerce, and utilities (Brown 2006). Examples that fall under the assertion of causing damage from a remote location could be that of an attacker opening a dam and releasing flood waters, causing a nuclear power plant meltdown, or causing an oil pipeline to burst (Brownlie 1963). Because these utilities are run on complex computer systems, there is a vulnerability that is easy for an attacker to penetrate and exploit (Weimann 2005). For this reason, the shift from traditional methods of attack to the more modern form of cyberterrorism is appealing because physical demands are diminished, the risk of death decreases, and the amount of time contributed by an attacker has less of a psychological effect. This, in turn, eases the burden for terror organizations to maintain the number of members dedicated to the cause (Weimann 2005).

Lastly, and most importantly, there is a media motivational aspect for attackers (Weimann 2005). As a concrete example of the motivation derived from media attention, in cases such as the I LOVE YOU virus, a virus that caused an estimated $10 billion in damages on 350,000 computers in over 20 different countries (Deal, Gage and Schueneman 2001), the media coverage garnered from that incident was larger in volume than could be expected had the incident occurred in one place (Subramanya and Lakshminarasimhan 2001). When each incident is covered with such depth by the media, an inflated sense of importance and meaning is attributed to each attack.

#### Cyberterrorism: a semiotic perspective

Cyberterrorist acts can be carried out through the Internet, a public communication channel. Cyberterrorism is publicized and propagated via new media communication. Consequently, it is fundamentally through semiotics and the exploitation of new media that cyberterrorists find success in achieving their chief goals. Semiotics is the study of signs (Berger 1989; Chandler 2002; Luskin 1996; Nöth 1995; Sebeok 1994). A sign is something that stands for something else or that can be created to represent something else (Deely 1990; Peirce 1934). The Internet is a sign system; it is an astronomical assemblage of codes and images thanks to which users can construct meanings and symbols. According to semioticians, humans do not face a “simple” objective reality. Rather, what humans see are signs and symbols within a communication framework, whereby the communication of messages is deemed quintessential to the creation of meaning (Fiske 1982). From this vantage point, meaning is not absolute; nor is it static. Meaning is an active process subject to constant transformation (Benford 1998).

Now that there is a foundation for understanding exactly what cyberterrorism is and the scope—both concrete and symbolic—it encompasses, a focus on the communicative aspect is warranted. It is not enough to know that these attacks are occurring. One must seek to uncover not only the method of communication, but also the meaning behind the communication. One note to mention when attempting to analyze the “intent” of another is the very concept of “intent.” When talking about motives, one must keep in mind that such a concept is intangible and as such will be immeasurable. As scholars who are not exactly certain of the exact motive behind the actions of an individual, we must examine overall behavior to tease out patterns and analyze the symbolic meaning behind those actions. In doing so, an understanding of propaganda is needed to place symbolic meaning in context.

### Uses of propaganda: general perspectives

Throughout the vast history of war, there have been many documented cases in which propaganda has been used as a catalyst for empowering terror organizations and providing them motivation for large-scale operations or attacks. By definition, propaganda is a mode of communication aiming at swaying the attitude of people toward some cause (Bernays and Miller 2004). For example, propaganda ignited motivation during wartime to increase membership in the armed forces (Lasswell 1971). It was also used as a means of trickery (Krippendorff and Bock 2008), as a way to or to gain a tactical advantage against the enemy (George 1959) or, most importantly, as a way to dehumanize the enemy by creating a realm of “the other” (Keen 1991). Verton (2003) explains that

al-Qaeda cells now operate with the assistance of large databases containing details of potential targets in the US. They use the Internet to collect intelligence on those targets, especially critical economic nodes, and modern software enables them to study structural weaknesses in facilities as well as predict the cascading failure effect of attacking certain systems (p. 109).

This Internet-based approach is considered postmodern, where the premise is that communication is directionless and leadership is not needed, nor does it exist (Matusitz 2008a, b). The Internet serves as the perfect medium for the trajectory of the modern terrorist: the cyberterrorist. While the tool (the Internet) has been indentified, previous research by Conway (2002) and Weimann (2006) shows that primary means of communication, intentional or otherwise, between cyberterrorist and their targets happen through a variety of employed propaganda. Jowell and O’Donnell (2006) state that “propaganda is the deliberate, systematic attempt to shape perceptions, manipulate cognitions, and direct behavior to achieve a response that furthers the desired intent of the propagandist” (p. 7).

The portrayal of the “other” (i.e., enemy) through propaganda is a method in which negative messages become continuously perpetuated. As such, the formation of in-groups occurs, which allows for beliefs and expectations to form and laws to emerge that dictate how the enemy is portrayed. Once these perceptions of an enemy form, they add motivation behind an attack (Keen 1991). When there is talk about “the other,” entire cultures become faceless, nameless, feeling-less entities that are the target of violence, and hate (Keen 1991). The language used in World War II propaganda consisted of “us” versus “them” mentality messages with terms such as “Commie bear,” “Nazi Swine,” and “Dog of Capitalism” (Keen 1991, p. 86), all of which dehumanize a given target. Because the use of propaganda is so powerful, it is important to understand how these various types of propaganda are effective, exactly what types are available for use and what is the driving force behind that power.

In regards to the question of power, Keen (1991) suggests that propagandist messages involve certain influential indicators that influence the subconscious psyche of a culture. To begin, it is essential to recognize the media as a strong and prominent outlet for terrorists to communicate propaganda (Cowen 2006). Another prominent medium in which propaganda is used as a means of communication is through the Internet (Hoffman 2003). A traditional method of terrorist communication previously employed was the use of video as a quick and effective method of relaying terrorist messages. In addition to the main focus of the use of video being a cheap and easy means of distributing propaganda for their cause, a more aggressive and destructive utilization of propaganda using the computer and Internet is through virus spreading (Weimann 2006). In the first half of 2005, documented worldwide cyber attacks from viruses reached a recorded 237, a 50 % increase from the same time period, 1 year earlier (Hoopes 2005).

Propaganda that follows the traditional model instructs an attacker to spend time effectively gathering intelligence on specific targets as a way to ensure that the maximum amount of damage that could possibly occur actually comes to fruition in each incident (Mathieu 2007). Certain tactics that are put into place start with extensive target analysis, intelligence gathering, and a network of command and control are considered necessities when attacking a target. All of these are designed to utilize many different directions to assault a target (Desouza and Hensgen 2003). The merging of traditional methods of attack with modern ones can be reflected in the way cyberterrorists pinpoint targets through the use of computers and by way of propaganda, recruitment, collection of data and information gathering, and member-to-member communication—through forums and videos via the Internet (Weimann 2006). An even more in-depth scope of these computer-based activities includes message posting, launching campaigns of a psychological nature, gathering information on potential targets, allowing for the synchronization of agendas and actions, allotting funds to specific areas, and using videos to conduct virtual terror training (Tzfati and Weimann 2002).

Continuing on with the understanding of the role of the media in current terrorist operations, it has been recognized that the media can manipulate and form desired images in respect to the minds of the public (Laqueur 2006). The example of the I LOVE YOU virus was a prime opportunity for media coverage on a massive scale. Such immense media coverage empowers terrorist organizations and provides motivation for continued attacks. Publicity and media are considered a necessity in the world of cyberterrorism, outlining two of the primary themes in the motivation of the attackers. Jenkins (1975) proposes that

propaganda terrorist attacks are often carefully choreographed to attract the attention of the electronic media and the international press. Taking and holding hostages increases the drama. The hostages themselves often mean nothing to the terrorists. Terrorism is aimed at the people watching, not at the actual victims. Terrorism is a theater (p. 4).

With the suggestion of the motives of terrorism rooted in theatrics, it is akin to suggesting that to be recognized in a highly visible and memorable way is the purpose for the attack, qualities that are often attributed to media coverage (Cowen 2006). What is meant by terrorism “as theater” or Debord’s (2005) terrorism “as spectacle” is not an exclusive activity reserved only for a selected group, rather a particular and precise display intended for an audience from one end of the spectrum to the other; much like a sporting event or a performance (Cowen 2006). These “theatrical” qualities—lack of regulation, easy access, vast range of audiences, and rapid information transfer—have allowed the goals of terrorists to be achieved, an increasingly attractive option when terror via the Internet allows for easy causing of damage with decreased fear of getting caught (Rogers 2003). Terrorist messages such as these are clearly heard worldwide due to well-developed and well-dispersed media contacts (Kim et al. 2002).

Similarly, Internet sites produce numerous opportunities for in-group communication and publicity, documenting a trend that encapsulates cause for organizations (Arquilla and Ronfeldt 2001a, b; Arquilla, Ronfeldt and Zanini 1999). The US State Department generated a list of terrorist organizations that confirmed that at least half of the known listed organizations have websites that are used for the solicitation of money and membership as well as a way for coded messages to make its way among group members (Gordon and Ford 2002). Internet provides the luxury of nonphysical contact with another member of the group where new recruits can become affiliated and commit to carrying out terrorist attacks, never actually leaving the comfort of home. In short, the use of propaganda has become the standard norm among terror groups (Harmon 2001).

Terrorist organizations require backing from supporters in the areas of both recruiting for membership and funding in order to continue to operate. Another use for propaganda is to discredit enemies (in the form of creating “the other”) all while placing the organizations in a positive light. Traditional propaganda techniques such as leaflets and publications in newspapers have now been replaced by the use of websites for financial backing and membership recruiting (Wright 1991). These leaflets and newspapers are truly an artifact of the past with the United States Department of State reported as early as 1999, that over one-third of the known Foreign Terrorist Organization (FTOs) had their own website (McGirk 1999).

### Uses of propaganda: examples of cyberterrorist groups

Popular radical groups of international significance such as Hezbollah, the Lebanese-based Shi’ite Islamic group (Conway 2002), operate Internet sites and use this outlet for various purposes such as posting articles or agendas of upcoming events, or to publish recently filmed videos, which can be accessed by anybody in the global cyber community (Deutsch 1996). Cyberterrorist organizations also feature disappearing and reappearing message boards and websites (Weimann 2006). One attacker, playing cat-and-mouse games with authorities through his websites, known as Irhabi007, emerged over the Internet as a leader of an online terrorist organization. His signature included online videos with instructions for home-made car bombs, and he also led forums criticizing American foreign policy, only to take them down and repost or list them under a different domain name (Fulghum 2005).

In November of 2005, as a tribute to a suicide bomber involved in the attacks on London, a full-length propaganda video entitled “The Martyrdom Will of Mohammad Sidique Khan” was posted by another terrorist group known as Sahaab—an arm of al-Qaeda—launched on the now-unresponsive website, www.as-sahaab.com. The video bore unassailable similarities to Irhabi007’s fundamental Islamist message board that had recently disappeared prior to the attack (Kohlmann 2006). Copycat websites playing the same cat-and-mouse games began to spring up after Irhabi007’s capture in 2005, with messages such as the following: “The enemies of Allah will continuously [try to close down] our website … We ask you to register for our mailing list so that you continue to receive the latest news of the Islamic Army in Iraq.” This post urged followers to continue their membership with the organization, despite seemingly inoperable websites (Kohlmann 2006).

Ultimately causing violent methods of destruction, Internet messages communicated between those cyberterrorist groups display consistent themes ranging from hate to anger (Talbot 2005). Attackers need a starting place. In order to inflict the most damage possible, an attacker needs to research various potential for damage in the process of building a target profile (Mathieu 2007). In order to utilize the Internet to its fullest extent, cyberterrorists can access a multitude of international areas and databases that contain sensitive information, such as libraries. Starting with access to legally obtained information, through legitimate search engines such as Google, attackers can gather information in the form of maps, satellite images, uploaded pictures and videos, and other texts available in seemingly harmless and innocent ways available in a public domain (Paul 2008). Browsing the Internet to gain information allows attackers to start building profiles against targets using simple resources that are also very much legal. Once the information-gathering process on a target has been completed and is recorded, an attacker can then use the Internet as a channel for carrying out the attack. The Internet, by way of computers, is the main tool available for assailants to coordinate and communicate on the method of attack (Paul 2008).

Encryption programs can be implemented to cover any harmful wrongdoing that could potentially be exposed throughout the course of the operation and, as this is being done, a system of hidden messages can be put into place (Paul 2008). Many of these messages range content-wise going so far to include instructions, step-by-step illustrated renderings of how an attack should be carried out, and detailed communicated plans enclosed in a secure network that requires a designated password to access. US Military computers have shown evidence of being a popular and frequent target by attackers. In 1998, cyberterrorists cracked into computers used by the Pentagon, using these methods of attack, and downloaded technical materials sensitive in nature (Lenzner and Vardi 2007). After a federal investigation, the source of the attacks proved to be a Moscow-based series of dial-up connections. The investigation, dubbed Moonlight Maze, was ineffective in catching the attackers.

The success of the terrorist group is directly correlated with keeping membership levels at a maximum, and as such, multiple methods of recruiting new members are a major focal point in the propaganda-based messages that are employed (Liu 2000). In past efforts to increase membership among groups, traditional methods of recruitment, such as published written work, audio–video tapes, CDs, and even local prayer leaders, have been employed as a means of promoting the cause (Paul 2008). The Internet, an updated and modern element of global terrorism, is emerging with websites and electronic forums that are used to spread ideological messages and provide hyperlinks between current operatives in cyberspace in addition to sharing graphic images depicting previous successes as a call to action for potential new members (Cronin 2006). In some instances, donations from sponsors or patrons are requested for those who wish to be supportive without being directly involved (Cronin 2006). The content of the websites offer a lesson on the history of the organization, and the cause the organization supports with the intent of enticing new members to join (Paul 2008). These websites also provide a venue for cyberterrorists to plan attacks by using a variety of methods that could not be achieved through other means.

The use of video provides another powerful arena utilized by terrorists. Video has been a vital part in the process of propaganda that is cheap and globally accessible (Weimann 2006). Films depicting anything from the morale-boosting success of radical fighters to the more macabre and disconcerting videos of executions, ambushes, and roadside bombings have emerged at a steady and continuous pace, being systematically distributed across the world (Kohlmann 2006). Terrorist group Zarqawi’s media chief, Abu Mayasara, displays the power of online videos when he posted, in a forum, an online insurgent video of high-ranking members of Zarqawi’s organization beheading American businessman Nicholas Berg (Glasser and Coll 2005). Mere weeks after that video was posted, additional copycat beheading videos trying to achieve the same gruesome effect as Zarqawi’s conquest and dozens of new unidentified Arabic-language message boards appeared rapidly on radical Islamist websites across the Internet (Kohlmann 2006).

The main difference in film distribution, to compare past methods to present day, is that in previous years, the videos, produced and distributed in traceable brick-and-mortar establishments, allowed for easy identification and easy prosecution of offenders, whereas present-day operations are postmodern and join Internet access with software designed for video editing and virtually untraceable upload capabilities (Kohlmann 2006).

In addition to easy access and virtual inability to be traced back to any one criminal, an appeal for the use of propaganda lies heavily in the ability to induce fear on a grand scale, affecting a multitude of people. Participants who were exposed to clips of terrorism and threats to national security developed higher anxiety than those who were not exposed to such clips, according to one study (Slone 2000). Perfidy or betrayal is an applicable outcome to the use of videos that rely on deceitful methods because of a reliance on outcomes that are psychologically damaging, allowing for a tactical advantage to be achieved (Dinstein 2004). Damaging and deceitful perfidy could be explained in a more detailed manner in regard to video, when the false construction or the blatant alteration of images or recordings occurs specifically to make a false claim against a party (Army Field Manual 1956). By extension, videos communicate a message to members of an organization and are used for purposes of displaying examples of previous successful attacks on a grand scale.

Another example of the deceitful nature in the form of damaging messages communicated through video comes to light when a multitude of videos are altered to express meaning that had not been originally intended (Slone 2000). Documented cases have exhibited modified and forged footage, such as falsely spliced voice recordings that depict an enemy head of state issuing orders for war crimes, or digitally altered state uniforms that have been changed to resemble enemy attire (Shulman 1999). Tactics such as these create consequences that are short term and steeped in deceit of a political nature. The consequences that occur long term—that of increased fatalities, extended periods of war, and schisms in the restoration of peace—destroy any foundation of peace that have been gained previously (Army Field Manual 1956). Additionally, propaganda allows for the perpetuation of “the other,” continuing the mindset of damaging nationalistic pride which “is the language of blood: a call to arms which can end in the horrors of ethnic cleansing” (Billig 1995, p. 48).

To date, evidence suggests that through means of technology—video, internet, and media coverage—messages through propaganda are worthy of mention because of the implications they carry from a communicative perspective. It has been suggested that restricted media coverage of terrorist attacks would in turn decrease the amount of terrorist attacks that occur afterward because a primary communicative intent—media coverage and recognition—was not being met (Cowen 2006). If this is the case, an interesting perspective to look for in the data would be the ties that connect the media, propaganda, and the communicative messages that are being conveyed.

### Combining semiotics and propaganda in a case study of cyberterrorism

This section provides a semiotic analysis of a case study of cyberterrorist propaganda and gives an explanation as to how the cyberterrorist act works both as a symbol and as terrorism. The case study focuses on a propagandist act committed by the World Fantabulous Defacers (WFD). This organization is a Middle-Eastern alliance of 12 cyberterrorist groups strongly opposed to the Indian presence in Kashmir and the occupation of Palestine by Israel. They have been reported in the news for wreaking havoc on websites (Aparna, Bolli and Bock 2008). In 2002, WFD hacked into the official website of Israeli Prime Minister Ariel Sharon and defaced it, causing thousands of dollars in damage. As a title, they wrote, “The Face of the World’s Biggest Murderer” (Verton 2003). They also inserted a dreadful picture of an injured Palestinian child and propagandist statements such as “Long Live Hizballah! Long Live Palestine! Long Live Chechnya, Kashmir, Kosovo, and Bosnia!” (Bunt 2003). At the bottom of the website, they incorporated a message with the signature of the group (Verton 2003).

The WFD’s hacking into Sharon’s official website illustrates the misdeed of a cyberterrorist group that had the capability to do far more damage and potentially create a national crisis in Israel (Verton 2003). Put simply, their misdeed constitutes a semiotic act encapsulated in messages and a horrific photo. By gaining such visibility, terrorists are now able to proliferate terror in cyberspace and evoke fear. While militant Palestinians blow up Israeli buildings, they can also use the Internet to cause harm to their enemies. This very attack was carried out by Palestinians sympathetic to their particular cause. Fear was generated and destruction was caused out of a political intent. The very act of defacing the Israeli Prime Minister’s official website may have caused thousands of dollars in damage, but, according to Bunt (1999, 2003), another objective of WFD was to diffuse Islamic supremacy.

It is fundamentally through semiotics and the exploitation of new media that the World Fantabulous Defacers found success in spreading propaganda. Semiotics is a tool to decode signs, their meanings and associations, and their evolution. The evolution, in this case study, is translated in a shift from traditional propaganda to e-propaganda (Karagiannis and Wagner 2007). Mandaville (2001) identifies a significant relationship between the Internet and Islamism. He points to the digitalization of Islamic terrorism. The Internet, it seems, has become an inseparable tool of Islamism. On the bright side, semiotics can also be an efficient tool for scholars and experts to detect and defeat cyber threats (Desouza and Hensgen 2005).

## Results and future directions

What this analysis has demonstrated is that cyberterrorists exploit diverse semiotic gestures, through the use of images and Internet videos, to communicate their intents to the public at large. In doing so, cyberterrorists communicate themes that range from hate to anger. From this vantage point, cyberterrorism is a form of theater or spectacle in which terrorists benefit from the endless opportunities that cyberspace offers to generate feelings of panic and overreaction in the target population. Cyberterrorism is a semiotic act; be it a message, a symbol, or an image on a website. Our computer-based universe is wrapped up with images, signs, and symbols. Truly, there is a powerful semiotic dimension to cyberterrorism.

So, through propagandist gestures and the use of various symbolic systems, cyberterrorists are capable of communicating their intents. The intent is to utilize any output necessary to play upon the fears to the public and by association, enhancing the power cyberterrorists wield. More specifically, this output is represented in coverage by the media generating increased attention and heightening the theatrical element behind each attack. Our society is wrapped up with images, signs, and symbols. Given this, there is a powerful semiotic dimension to cyberterrorism. Without a doubt, it can involve sending images of fear. We saw it with the cyberterrorist act committed by the World Fantabulous Defacers (WFD) in 2002. It is essentially by means of semiotics and the utilization of new media that WFD managed to successfully spread their propagandist messages. In like fashion, the full-length propaganda video entitled “The Martyrdom Will of Mohammad Sidique Khan” was posted by another terrorist group known as Sahaab—an arm of al-Qaeda—launched on the now-unresponsive website, www.as-sahaab.com. The creators of the video had one goal in mind: to instill feelings of panic in viewers, through powerful images (Kohlmann 2006).

Also demonstrated is a carefully crafted network of Internet savvy members of cyberterrorist organizations who communicate power and status through online video clips, websites, and through methods of destruction ranging from the malicious (denial of service) to the irreparably devastating (death). The motives of cyberterrorists are the same as those of conventional terrorists: to send images of fear. In the same way that terrorism is, first and foremost, a process of communication between terrorists and target audiences (Tuman 2003), a key objective of cyberterrorists is as old as the one by conventional terrorists: to send a powerful signal whose meaning is intended to frighten and to coerce.

The interesting notion, as mentioned before with the cat-and-mouse nature of Islamist cyberterrorist Irhabi007 (Kohlmann 2006), is that these terrorist websites are frequently put up and taken down so they can cause their damage and still be maintained for another day. The general scope for the use of websites is so vast that they provide a forum, or a safe haven for any level of content that a cyberterrorist feels is necessary to air to keep motivation for the cause intact, for reasons of member recruitment or to raise funds from supporters. While the primary goal of terrorism is a process of communication between terrorists and target audiences (Tuman 2003), cyberterrorism also seeks to send a powerful signal meant to frighten and coerce the target. This analysis detailed the various motivations behind small- and large-scale targets and the emotional aspects of fear for safety and lack of faith in the government that accrues from being targeted.

For future research, it might prove interesting to continue investigating the relationship between cyberterrorism and new media (i.e., Internet and other information technologies). Without the existence of these, cyberterrorism is doomed to failure. In fact, scholars should examine the two following questions: How different would cyberterrorism be without semiotics? And what would cyberterrorism be without Internet-facilitated propaganda? The use of communication technologies by cyberterrorists is an essential requirement for the success of their propagandist and semiotic gestures. In order to cause massive overreaction from the public, cyberterrorists rely on those new media to agitate the target population by exploiting images that, once produced, can be exploited again later and be re-used to new effect.

As we can see, cyberterrorism represents a mighty tool of communication, persuasion, and propaganda. Since billions of human beings are becoming increasingly interconnected through computers and the Internet, cyberspace creates both benefits and disadvantages for human communities. The danger of cyberterrorism is real; though it has been underestimated by many, it can add a great deal to our anxieties.
